# Detection of EGFR Mutations From Plasma of NSCLC Patients Using an Automatic Cartridge-Based PCR System

**DOI:** 10.3389/fphar.2021.657743

**Published:** 2021-04-14

**Authors:** Simon Heeke, Véronique Hofman, Jonathan Benzaquen, Josiane Otto, Virginie Tanga, Katia Zahaf, Maryline Allegra, Elodie Long-Mira, Sandra Lassalle, Charles-Hugo Marquette, Marius Ilie, Paul Hofman

**Affiliations:** ^1^ Department of Thoracic H&N Medical Oncology, UT MD Anderson Cancer Center, Houston, TX, United States; ^2^ Laboratory of Clinical and Experimental Pathology, Biobank BB-0033-00025, Centre Hospitalier Universitaire Nice, Nice, France; ^3^ Team 4 IRCAN, Inserm U1081/CNRS 7284, IRCAN, Nice, France; ^4^ FHU Oncoage, Nice, France; ^5^ Pulmonary Department, Pasteur Hospital, Nice, France; ^6^ Department of Medical Oncology, Centre Antoine Lacassagne, Nice, France

**Keywords:** EGFR, liquid biopsy, idylla, cobas, plasma, circulating tumor DNA

## Abstract

The introduction of liquid biopsies for the detection of *EGFR* mutations in non-small cell lung cancer patients (NSCLC) has revolutionized the clinical care. However, liquid biopsies are technically challenging and require specifically trained personnel. To facilitate the implementation of liquid biopsies for the detection of *EGFR* mutations from plasma, we have assessed a fully automated cartridge-based qPCR test that allows the automatic detection of *EGFR* mutations directly from plasma. We have analyzed 54 NSCLC patients and compared the results of the cartridge-base device to an FDA-approved assay. Detection of EGFR mutations was comparable but slightly lower in the cartridge-based device for L858R mutations (14/15 detected, 93%) and exon 19 deletions (18/20 detected, 90%). Unfortunately, 8/54 (15%) tests failed but increasing the proteinase K volume helped to recover 3/4 (75%) unsuccessful samples. In summary, the fully automated cartridge-based device allowed the detection of EGFR mutations directly from plasma in NSCLC patients with promising accuracy. However, protocol adjustments are necessary to reduce a high test failure rate.

## Introduction

The introduction of liquid biopsies (LB) for the detection of Epidermal growth factor receptor (*EGFR*) mutations at diagnosis as well as at tumor progression during treatment to identify resistance to tyrosine kinase inhibitors (TKIs) treatment has revolutionized care of non-small-cell lung cancer (NSCLC) patients (Oellerich et al., 2019). Today, several assays using either qPCR (such as the Cobas or Therascreen assays) or Next Generation Sequencing (NGS) [such as the FoundationOne Liquid CDX (Foundation Medicine, Cambridge, United States) assay or the Guardant 360 assays (Guardant Health, Redwood City, United States)] are approved in the United States and many other countries for the detection of *EGFR* mutations and are used in routine clinical care (Aggarwal et al., 2021). However, while LB demonstrated an improved turn-around time (TAT) compared to tissue biopsy testing, the time needed to generate results in routine, notably when using NGS, still requires several days and is mainly limited by batching (the time until sufficient samples are gathered to start a batch of analysis). The need to wait for a sufficient number of samples to start an analysis run can be overcome by using outsourced tests which in turn require additional time for the sending to the certified testing centers which can have a significant impact on the TAT (Heeke et al., 2019). In-house tests in contrast are usually labor intensive and may require special training but are often faster and less expensive than outsourced tests (Mrak et al., 2018). Here we assessed a fully automated test that generates reports on *EGFR* mutations directly from plasma by automatizing all the necessary steps from DNA extraction, PCR and report generation in a cartridge-based design in which each sample is run independently. Interestingly, this assay is no longer limited by batching and is able to generate results within 3 h with minimal hand-on time and with minimal training requirements. This would consequently dramatically reduce TAT for LB with same-day-reporting. Therefore, we wanted to compare the diagnostic performance of the novel cartridge-based PCR system to a CE-IVD and FDA approved test by assessing specificity, sensitivity and clinical implementation. This would allow for the first time the easy and fully automatic assessment of *EGFR* mutation detection from plasma of non-small cell lung cancer patients.

## Methods

### Patient Inclusion and Sample Selection

For the analysis, 54 advanced NSCLC patients were retrospectively included. *EGFR* status was obtained from primary tissue samples obtained at diagnosis which were tested using the CE-IVD Idylla EGFR assay that is based on the same principle as the here described ctDNA assay. Analysis for those tissue sections has been performed as described previously (Ilie et al., 2017). Patients were selected to represent a broad range of clinically relevant *EGFR* mutations aiming to include 30% exon 19 deletions (Del19), 30% L858R mutations and 10% of patients with other mutations. Plasma isolation has been performed as reported previously (Heeke et al., 2020). Additionally, 30% of all patients tested were aimed to be *EGFR* wild type serving as control. All patients provided written informed consent and the study was in accordance to the declaration of Helsinki.

### Cartridge-Based Mutation Detection

The Idylla ctEGFR Mutation Assay is a research-use only (RUO) qPCR-based test that automatizes all analysis steps from DNA extraction to final report generation using a single-use cartridge system (Biocartis, Mechelen, Belgium). The cartridge consists of a loading chamber in which the sample is added and after entering the cartridge in the Idylla system, the isolation of DNA as well qPCR using fluorescence dyes is automatically performed in individual chambers in the cartridge which also contains all the reagents (Van Haag et al., 2006).

The assay covers 49 *EGFR* mutations in exons 18, 19, 20, and 21 (Supplementary Table S1). For the analysis, 2 ml of plasma together with 20 μl proteinase K (20 mg/ml, Thermo Fisher Scientific, Waltham, United States) was directly pipetted in the cartridge which was loaded in the Idylla system performing all steps automatically in approximatively 3 h TAT. One assay could be loaded per cartridge, but the device was able to run several cartridges in parallel.

### FDA Approved PCR-Based Mutation Detection

For the analysis of concordance, the samples were also tested with the Cobas *EGFR* Mutation Test v2 (Roche, Basel, CH) as reported previously (Heeke et al., 2020). Importantly, this qPCR-based assay is approved by the FDA for the detection of *EGFR* mutations from plasma samples and is certified by ISO 15189 in our laboratory (www.cofrac.fr).

### Data Analysis

Samples that were discordant between the two assays were additionally tested using Stilla digital PCR (Stilla technologies, Villejuif, FR) as reported previously (Heeke et al., 2020). This was only possible for samples with sufficient DNA left and for samples with exon 19 deletions or L858R mutations as our assay is not supporting any additional primary mutations (but covers the T790M resistance mutation).

Data was analyzed using R v.4.0.1 (R Foundation for Statistical Computing, Vienna, Austria). Sensitivity, specificity and 95% CI intervals were calculated using MedCalc.

## Results

In total, 54 samples were tested using the automated cartridge-based PCR system of which 46/54 (85%) were conclusive giving an interpretable result. Two additional samples were repeated leading to 48 successfully analyzed samples in total (89%). Failure of obtaining a result was due to a technical error that led to blocking of the cartridge by the plasma during the sample processing and which prevented to obtain PCR results. For four additional samples with sufficient plasma left, we re-run the cartridge-based system this time with 200 μl of proteinase K instead of the previously used 20 μl and we were able to retrieve results for 3/4 (75%) samples (Supplementary Figure S1).

**FIGURE 1:**
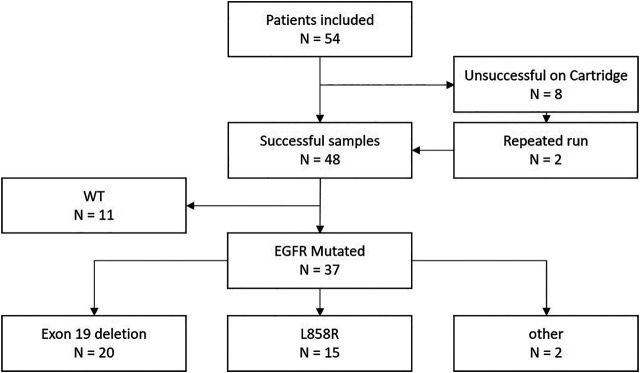
Flowchart of the study.

Of the 48 initially successfully samples on the cartridge-based system, 11 samples were WT for *EGFR* mutations and 37 presented with an EGFR mutation (Figure 1).

**FIGURE 2:**
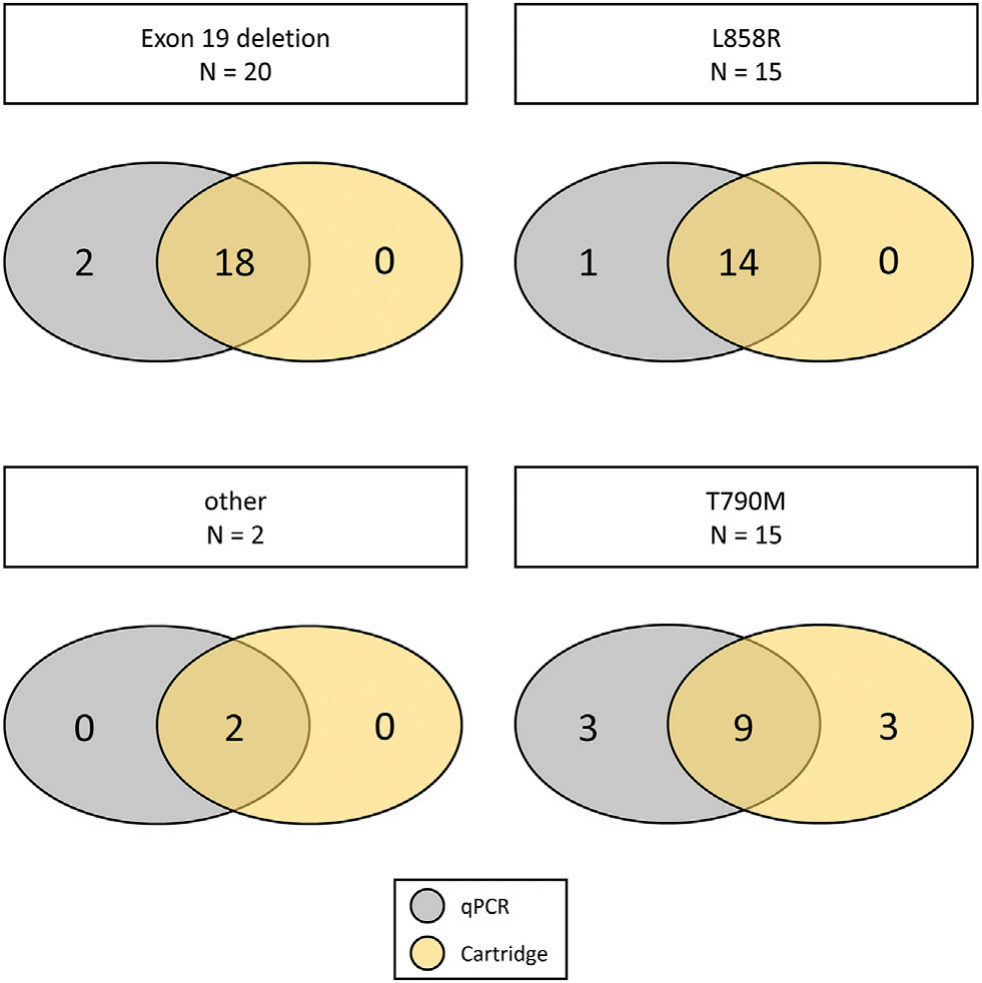
Overview on the mutations detected by the different systems. Each row represents one of the systems used for the detection of *EGFR* mutations and each case is represented by one column.

**FIGURE 3:**
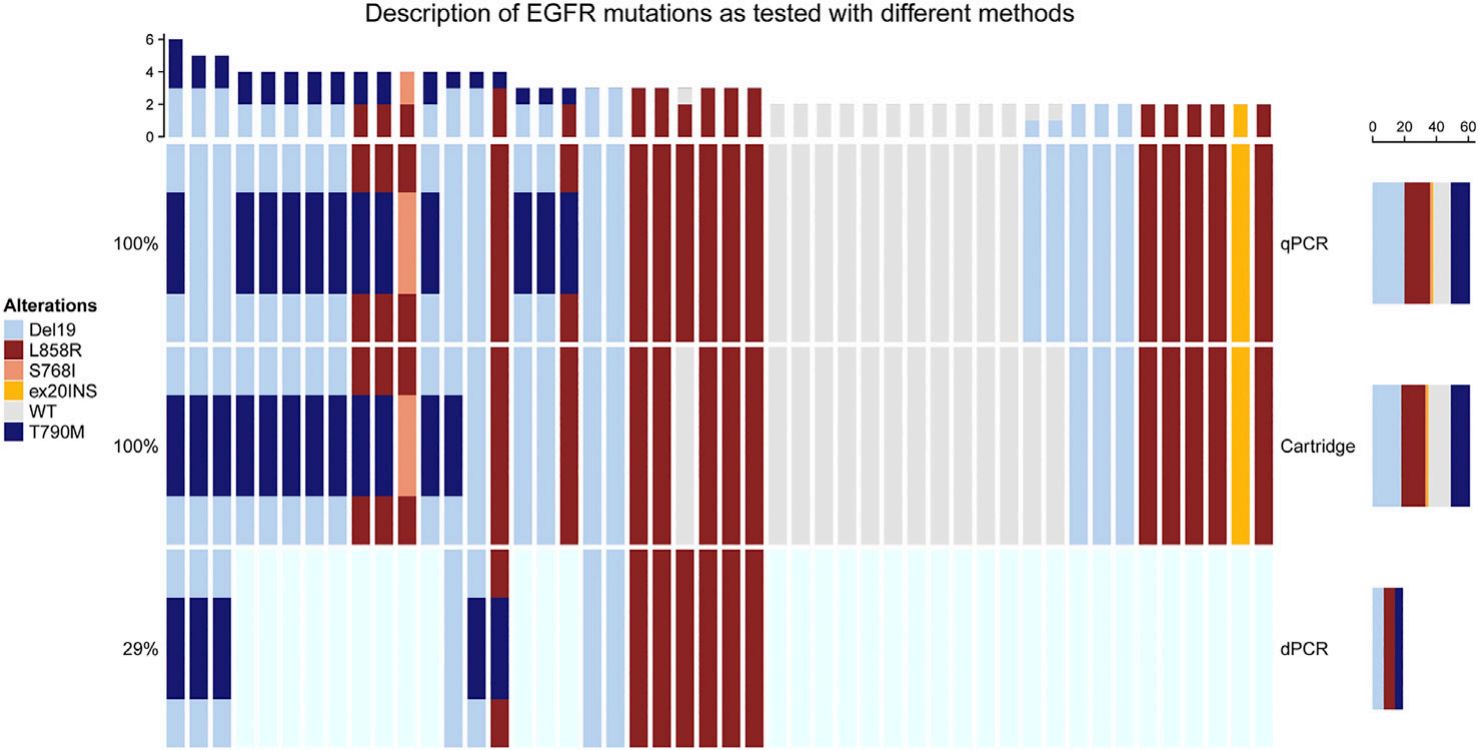
Concordance of mutations detected by the cartridge-based system in comparison to the FDA approved qPCR based system for each of the mutation types. The number of samples with the respective mutations is shown in the middle if concordant, on the right side if only detected in the cartridge-based system or on the left side when only detected with the qPCR based system.

Exon 19 deletions (*N* = 20) and L858R mutations (*N* = 15) were the most frequent mutations detected. Additionally, one S768I and L858R compound mutation as well as one insertion in Exon 20 were detected (Figures 1, 2). Concordance of the automated cartridge-based PCR system with the CE-IVD qPCR test was very good for all the mutations, however, 2/20 (10%) exon 19 deletions and 1/15 (7%) L858 R mutations remained undetected on the cartridge-based system (Figure 3).

Consequently, the sensitivity of the cartridge-based system was 90.91% (95% CI: 70.84–98.88%) for exon 19 deletions and 93.75% for L858R (95% CI: 69.77–99.84%), respectively. For the T790M resistance mutation, both systems failed to detect 3/15 (21%) mutations. All those mutations were confirmed using a digital PCR (dPCR) system. Additionally, one T790M mutation was only detected using the dPCR system and remained undetected on both systems highlighting a sensitivity of 75% for both systems compared to dPCR (95% CI: 47.62–92.73%). Specificity for all mutations tested was 100%.

Interestingly, for all mutations that were not detected using the cartridge-based system, a signal was detected for the discordant mutation which however remained below the pre-specified threshold to call the respective mutation as exemplified in Supplementary Figure S1.

Finally, a direct comparison of costs is dependent on many variables, like country, ability to negotiate, cost of personnel and sample throughput. However, by calculating approximate costs in our clinical setting, we can consider the costs between the cartridge-based system and the FDA approved system very comparable and thus costs should not be a factor when considering which test to implement in routine clinical care.

## Discussion

Running a LB test for the detection of *EGFR* mutations in a fully automatized was feasible in our study. The reduced hands on time dramatically reduced the time needed by the personnel improving the efficacy of resources in routine daily use. Importantly, the simple cartridge-based design allows the fast implementation in laboratories that are already used to the system with minimal training allowing the easy implementation of LB in laboratories which have previously restrained from the investment. Additionally, as one cartridge is loaded at a time and as there is no need for cfDNA extraction, there is minimal risk of handling errors and of contaminating the samples with other sources.

While the specificity in the present cohort was 100% for all the mutations tested, the sensitivity for primary mutations was reduced compared to the FDA approved qPCR system with one L858R and two exon 19 deletions missed on the cartridge-based system. Interestingly, the detection of secondary T790M mutations was equivalent to the FDA-approved test with both systems having missed three of the mutations. The cartridge-based system has the possibility to directly access the amplification curves via the software Idylla Explore, which revealed an amplification for all the missed mutation which were however below the prespecified threshold. Consequently, adjusting the threshold or improving the workflow might indeed increase the sensitivity of the assay yielding results that are equivalent to the FDA approved reference test. Additionally, this might give the possibility to evaluate if re-analyzing of a sample might be advised to correctly detect mutations that were so far below the pre-specified threshold. However, adjusting the threshold always poses the risk of reducing specificity and thus would require evaluation in a larger prospective study to not impair the performance of the assay. Interestingly, based on a study made with previously isolated DNA from FFPE tissue specimen, the CQ value of the internal control of the cartridge-based system could indicate when a reanalysis of the results would be recommended, which could overcome uncertainties when analyzing mutations which were below the predefined threshold (Grant et al., 2021). The recent development of NGS systems with fast turn-around times increasingly challenges the use of single-gene assays (Low et al., 2020). However, the NGS workflow still requires batching of samples and increased hands-on time. Additionally, a recent prospective trial analyzing different testing methods for *EGFR* mutations from plasma confirmed the high reproducibility across PCR-based and NGS-based platforms demonstrating the relevance of PCR-based assays for single-gene mutation testing in routine clinical care (Romero et al., 2021). Lastly, it is important to note that the ctDNA concentrations in plasma are dependent on tumor burden, site of metastasis but also genotype and *EGFR* gene amplifications which might impair performance of the *EGFR* mutation detection form plasma (Lam et al., 2020). However, those limitations are independent of the technique used.

A major limitation of the cartridge-based system remained the high number of failed runs 8/54 (15%). It seems that the viscosity of the plasma samples blocked some of the internal chambers of the cartridge which prevented the successful qPCR amplification. Adjusting the protocol to a higher amount of proteinase K which should decrease viscosity by degrading the protein content of plasma has resolved this error (in 75% of the cases tested). Therefore, we would highly recommend to generally increase the amount of 200 μl proteinase K compared to the amount of 20 μl initially used in this study but also to the 30 µl amount recommended by the manufacturer. Nevertheless, this limitation needs certainly additional assessment especially in larger studies to correctly analyze how the error rate can be reduced to a clinically acceptable rate as the error rate observed in this study was certainly too high to recommend its implementation in clinical routine use. Additionally, like all PCR-based assays, the cartridge-based assay is limited by the selection of pre-specified mutations. While all mutations for which an FDA/EMA approved therapy exist are included, as well as the T790M resistance mutations that requires the administration of a third generation TKI, other mutations which might become relevant might be missing (Planchard et al., 2018). Most prominently, the C797S mutation that was described to mediate resistance to a third generation TKI, is lacking but might certainly be included in later, updated versions of the assay (Thress et al., 2015).

In conclusion, the fully automatized and cartridge-based system demonstrated a promising diagnostic performance with the potential to be equivalent to another previously FDA-approved assay while a significant error rate in the cartridge-based system needs further attention, for example protocol adjustments that reduced this error in our analysis. Importantly, the fast TAT, low hands-on-time, easy implementation and low training requirements would allow the implementation of plasma-based *EGFR* detection in a short time thereby enabling the administration of the appropriate treatment in NSCLC patients.

## Data Availability

The raw data supporting the conclusions of this article will be made available by the authors, without undue reservation.
