# Supplementary material for: Detection of EGFR Mutations From Plasma of NSCLC Patients Using an Automatic Cartridge-Based PCR System
Source: Front Pharmacol. 2021 Apr 14;12:657743. doi: 10.3389/fphar.2021.657743 (PMC8079969; doi:10.3389/fphar.2021.657743)
Supplement: Supplementary file 1 [file datasheet1.docx]

Supplementary Material

**Supplementary Table 1:** Mutations covered by the cartridge-based qPCR assay.

| Exon | Mutation | Mutation (Amino Acid) | Mutation (DNA) | Result Report |
| --- | --- | --- | --- | --- |
| Exon 18 | G719A  G719C  G719C  G719S | p.Gly719Ala  p.Gly719Cys  p.Gly719Cys(2)  p.Gly719Ser | c.2156G>C  c.2155G>T  c.2154_2155delinsTT  c.2155G>A | G719A/C/S |
| Exon 19 | Del9 | p.Leu747_Ala750delinsPro  p.Leu747_Ala750delinsSer p.Leu747_Glu749del | c.2238_2248delinsGC c.2239_2248delinsC  c.2240_2248del  c.2239_2247del | Exon 19 deletion |
|  | Del12 | p.Leu747_Thr751delinsPro  p.Leu747_Thr751delinsSer | c.2239_2251delinsC  c.2240_2251del |  |
|  | Del15 | p.Glu746_Ala750del  p.Leu747_Thr751del  p.Glu746_Thr751delinsAla p.Glu746_Thr751delinsIle p.Glu746_Thr751delinsVal p.Lys745_Ala750delinsThr p.Glu746_Thr751delinsLeu p.Glu746_Thr751delinsVal p.Glu746_Thr751delinsAla p.Glu746_Thr751delinsGln  p.Ile744_Ala750delinsValLys | c.2235_2249del c.2236_2250del  c.2239_2253del  c.2240_2254del  c.2238_2252del  c.2237_2251del  c.2237_2251del  c.2235_2252delinsAAT  c.2237_2252delinsT  c.2234_2248del  c.2236_2253delinsCTA  c.2237_2253delinsTA  c.2235_2251delinsAG  c.2236_2253delinsCAA c.2230_2249delinsGTCAA |  |
|  | Del18 | p.Leu747_Pro753delinsSer p.Glu746_Ser752delinsVal p.Leu747_Ser752del  p.Glu746_Thr751del p.Leu747_Pro753delinsGln p.Glu746_Ser752delinsAla p.Glu746_Ser752delinsAsp p.Glu746_Ser752delinsIle  p.Glu746_Ser752delinsVal | c.2240_2257del  c.2237_2255delinsT  c.2239_2256del  c.2236_2253del  c.2239_2258delinsCA  c.2237_2254del  c.2238_2255del  c.2236_2256delinsATC  c.2237_2256delinsTT  c.2237_2256delinsTC  c.2235_2255delinsGGT |  |
|  | Del21 | p.Leu747_Pro753del  p.Glu746_Ser752del | c.2238_2258del  c.2236_2256del |  |
|  | Del24 | p.Ser752_Ile759del | c.2253_2276del |  |
| Exon 20 | T790M | p.Thr790Met | c.2369C>T | T790M |
|  | S768I | p.Ser768Ile | c.2303G>T | S768I |
|  | InsG InsASV9 InsASV11  InsSVD  InsH | p.Asp770_Asn771insGly p.Val769_Asp770insAlaSerVal p.Val769_Asp770insAlaSerVal p.Asp770_Asn771insSerValAsp  p.His773_Val774insHis | c.2310_2311insGGT  c.2307_2308insGCCAGCGTG c.2309_2310delinsCCAGCGTGGAT c.2311_2312insGCGTGGACA  c.2319_2320insCAC | Exon 20 insertion |
| Exon 21 | L858R | p.Leu858Arg | c.2573T>G  c.2573_2574delinsGT c.2573_2574delinsGA | L858R |
|  | L861Q | p.Leu861Gln | c.2582T>A | L861Q |


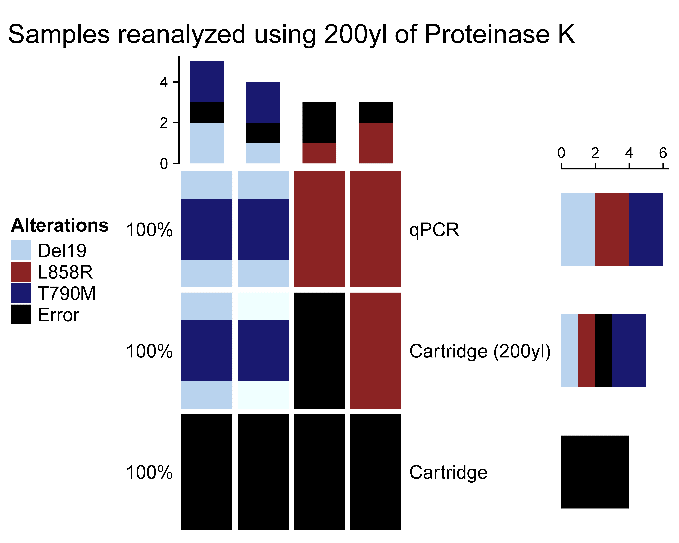


Supplementary Figure 1: Overview on the mutations detected by the different systems for the four samples that were retested with 200μl of Proteinase K. Each row represents one of the systems used for the detection of EGFR mutations and each case is represented by one column.
